# Validation of DTI Tractography-Based Measures of Primary Motor Area Connectivity in the Squirrel Monkey Brain

**DOI:** 10.1371/journal.pone.0075065

**Published:** 2013-10-01

**Authors:** Yurui Gao, Ann S. Choe, Iwona Stepniewska, Xia Li, Malcolm J. Avison, Adam W. Anderson

**Affiliations:** 1 Department of Biomedical Engineering, Vanderbilt University, Nashville, Tennessee, United States of America; 2 Institute of Imaging Science, Vanderbilt University, Nashville, Tennessee, United States of America; 3 Department of Psychology, Vanderbilt University, Nashville, Tennessee, United States of America; 4 Department of Radiology and Radiological Sciences, Vanderbilt University, Nashville, Tennessee, United States of America; 5 Department of Pharmacology, Vanderbilt University, Nashville, Tennessee, United States of America; 6 Department of Neurology, Vanderbilt University, Nashville, Tennessee, United States of America; University of Alberta, Canada

## Abstract

Diffusion tensor imaging (DTI) tractography provides noninvasive measures of structural cortico-cortical connectivity of the brain. However, the agreement between DTI-tractography-based measures and histological ‘ground truth’ has not been quantified. In this study, we reconstructed the 3D density distribution maps (DDM) of fibers labeled with an anatomical tracer, biotinylated dextran amine (BDA), as well as DTI tractography-derived streamlines connecting the primary motor (M1) cortex to other cortical regions in the squirrel monkey brain. We evaluated the agreement in M1-cortical connectivity between the fibers labeled in the brain tissue and DTI streamlines on a regional and voxel-by-voxel basis. We found that DTI tractography is capable of providing inter-regional connectivity comparable to the neuroanatomical connectivity, but is less reliable measuring voxel-to-voxel variations within regions.

## Introduction

Diffusion Tensor Imaging (DTI) is a noninvasive method to characterize the microstructure of biological tissue [1]. It is based on measurements of the mean squared displacement of water molecules along predetermined directions, estimated from the signal decay in a pulsed gradient spin echo acquisition [2]. Water diffusion is anisotropic in many tissues, especially in fibers of tightly packed, parallel axons [3] in brain white matter. Hence, DTI serves as a sensitive probe of axonal fiber orientation [4]. Based on these concepts, white matter pathways can be reconstructed by tracking the inferred axonal orientations step by step (with voxel or subvoxel step size) from seed points. Deterministic tracking algorithms [5]–[9] construct a unique path from each seed point whereas probabilistic algorithms [10]–[14] generate multiple possible paths from each seed point. Both families of tractography algorithms have become valuable research tools for the *in vivo* study of neuronal tissue morphology, pathway location, and other properties which directly relate to neuropsychiatric/neurologic disorders [15], brain organization and development [16], [17].

Among many potential applications of DTI tractography, mapping anatomical connectivity [18] has attracted the attention of many neuroscientists, neurologists, and psychiatrists, since knowing the network structure within and between brain regions is a fundamental prerequisite for understanding mechanisms underlying brain functions and dysfunctions. Early experimental methods to reveal connectivity employed gross dissection [19] and neural degeneration procedures [20]–[22]. More recent experiments exploit neural tracers, which capitalize on cytoplasmic flow and the axoplasmic transport system [23]. A variety of tracers, such as horseradish peroxidase (HRP), lectins, toxins and their conjugates, fluorescent dyes, dextrans and viruses (for review see [24]), have been used in countless investigations that have contributed valuable descriptions of connectivity in the mammalian brain. These methods, however, are highly invasive and require fixed, processed tissue for data analysis, preventing their use for *in vivo* connectivity mapping. The recently introduced manganese technique offers an opportunity to study neuronal connectivity *in vivo* by means of magnetic resonance imaging (MRI) [25], but the technique has several drawbacks that can reduce its applicability, the most important being the potential toxicity of the Mn^2+^ ions.

DTI tractography overcomes the problem of invasiveness, but whether or to what extent it reveals true anatomical connectivity is unclear. A number of well-examined factors, e.g., signal to noise ratio [26] and partial volume effects [27], may degrade the fidelity with which tractography can represent anatomical connectivity [28]. Hence, the agreement between tractography-derived and anatomical connectivity needs to be evaluated quantitatively. Previous *in vivo* or *in vitro* validation studies [29]–[31] mainly compare tractography-derived pathways with the ‘ground truth’ white matter fiber bundles revealed using surgical dissection, neural tracer tracking or an MR-visible tracer. They demonstrated general agreement in a number of specific bundles between tractography-derived pathways and the ‘ground truth’. Besides the morphology of white matter pathways, there is increasing interest in measuring cortico-cortical connectivity using DTI tractography [32]. However, to the best of our knowledge, such measurements have not yet been rigorously validated.

This paper aims to assess the accuracy of DTI tractography as a measure of cortico-cortical connectivity. For this purpose, we studied neuroanatomical and DTI tractography-derived cortico-cortical connections in an animal model and quantitatively evaluated the histology-tractography agreement based on regional and voxelwise analysis.

## Methods

### Tracer Injection

The experiment was carried out on a New World squirrel monkey. The brain of this species shares many features of functional organization and microstructural complexity with humans, although the cortical surface is less gyrated. The motor cortex, for example, is completely exposed on the brain surface, so it is easily accessible for electrical microstimulation and injection of tracers. Other advantages of New World monkeys are that the cortical connections of the primary motor cortex (M1) are well known from previous studies [33] and their smaller brains reduce the time of histological processing and anatomical data analysis.

**Figure 1:**
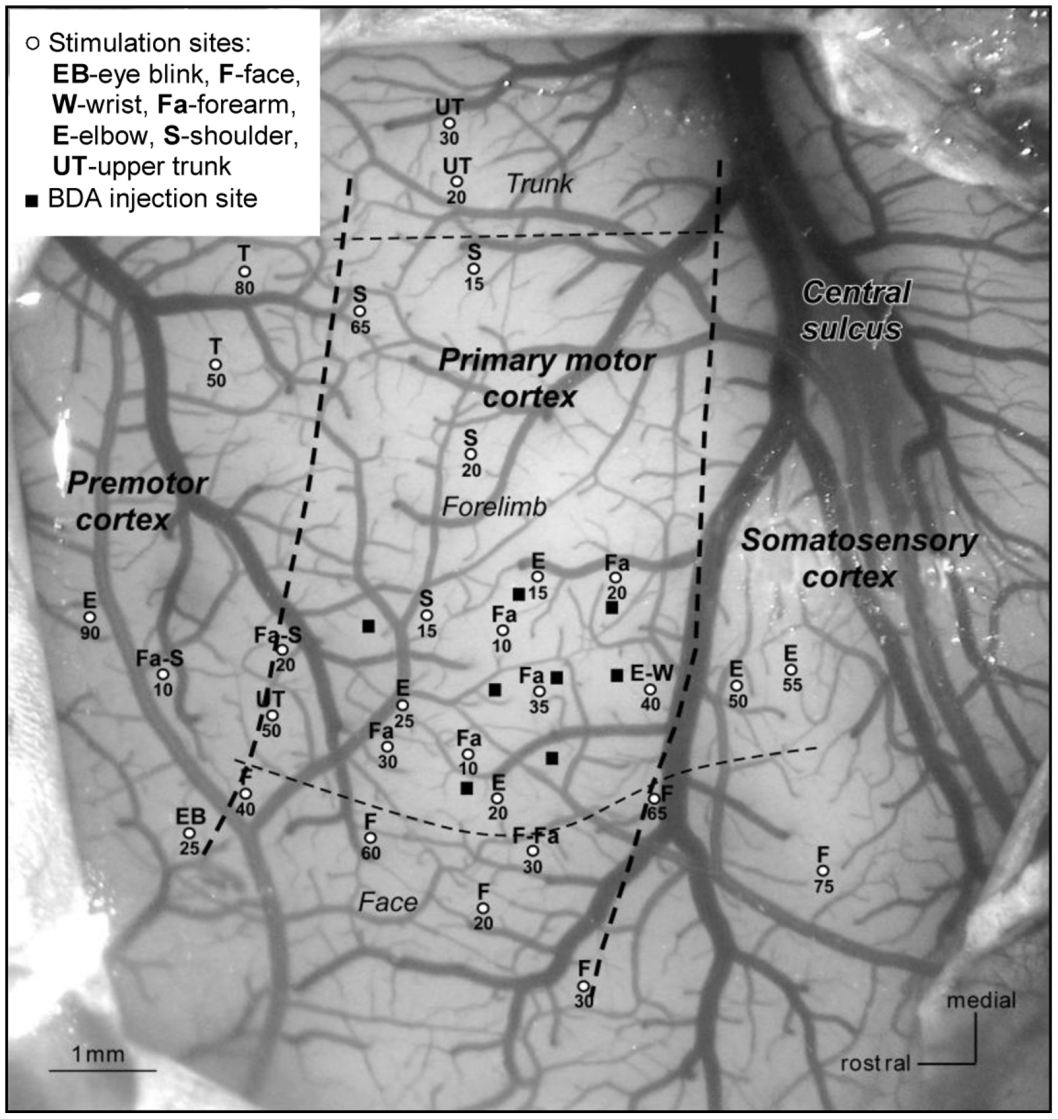
Functional map of the primary motor cortex (M1) used to guide BDA injections. Cortical sites marked as white dots were stimulated by micro electrode, which evoked body movements of the anesthetized monkey. Letter(s) above each dot indicate the specific movement(s) corresponding to this stimulation site. The number below each dot represents the current threshold (in units of µA) needed to evoke the movement. Thick dashed lines indicate approximate borders of M1, which were identified by the magnitude of threshold (thresholds lower than approximately 40 µA were used to infer the M1 region). Thin dashed lines indicate approximate borders between M1 body representation areas. Black squares show the BDA injection sites covering the forearm movement representation area.

Under general anesthesia using aseptic techniques, a bidirectional tracer, biotinylated dextran amine (BDA; Molecular Probes Inc., Eugene, OR) was injected (as a 10% solution in phosphate buffer) into left hemisphere M1. Pressure injections of BDA were carried out using a 2 ul Hamilton syringe. Eight injections (1 µl/site; indicated by the black squares in Fig. 1) were made to cover a large M1 region representing the forearm and identified by intracortical microstimulation (see the stimulation sites labeled ‘Fa’ in Fig. 1). After each injection, the needle was left in the brain for 5–10 minutes and then retracted stepwise to avoid leakage of the tracer along the needle track. Surgical, microstimulation and injection procedures have been described in detail elsewhere [33]. After surgery, the monkey was allowed to recover from the procedure, giving the tracer sufficient time to be transported along axons to all regions connected to M1.

All animal procedures were approved by the Vanderbilt University Animal Care and Use Committee, and followed guidelines of National Institutes of Health for the care and use of laboratory animals. The monkey was housed in adjoining individual primate cages allowing social interactions with other monkeys and received extensive environmental enrichment such as play objects that are changed for variability, novel food objects and foraging devices. Normal food, treats and fruit were provided to the animal by trained staff. Analgesics were given following the procedure and in response to any evidence of pain (behavioral abnormality, loss of appetite, tissue edema, or muscle spasms). The monkey was closely monitored in the week following the surgical procedures.

### Ex vivo MRI Data Acquisition

One week after surgery, the monkey was given a lethal dose of barbiturate, and perfused through the heart. All blood was rinsed out with physiological saline (0.9% NaCl) followed by fixative (4% paraformaldehyde). The brain was removed from the skull and stored in buffered saline overnight. The next day, the brain was scanned on a 9.4 Tesla Varian scanner (Varian Inc., Palo Alto, CA). First, T2-weighted structural images were acquired by running a standard gradient echo multi-slice (GEMS) sequence with full brain coverage (TR = 963 ms, TE = 4 ms, flip angle = 20°, slice gap = 0 mm, voxel size = 300×300×300 µm^3^, data matrix = 128×128×192, SNR≈50). Then diffusion weighted imaging was performed using a pulsed gradient spin echo (PGSE, [2]) multishot spinwarp imaging sequence with the same FOV as the structural images (TR = 5.2s, TE = 26 ms, number of diffusion gradient directions = 31, *b* = 0, 1200s/mm^2^, voxel size = 300×300× 300 µm^3^, data matrix = 128×128×192, number of acquisitions = 10, SNR≈25, scanning time≈50 hr). The *b* value used in this experiment was lower than is optimal for diffusion studies in fixed tissue [34], due to hardware limitations. A low *b* value decreases the available diffusion contrast-to-noise ratio (CNR) in the image data, which has the same effect as higher image noise. To compensate for this shortcoming, we extended the scan time to 50 hours, which yielded a CNR comparable to *in vivo* human studies (equivalent to an *in vivo* study with mean diffusivity = 0.7×10^−3^ mm^2^/s and SNR≈20).

### Block/micrograph Data Acquisition

Following MRI scanning, and before sectioning, the entire brain was placed in 30% sucrose for cryoprotection. Two days later the brain was cut serially on a microtome in a coronal plane to produce 50 µm thick frozen sections. All sections were collected in phosphate buffer, but prior to cutting every third section (i.e., at 150 µm intervals), the frozen tissue block was photographed using a Canon digital camera (image resolution = 50 µm/pixel, image size = 3330×4000pixels, number of images = 286), mounted above the microtome to facilitate intermodality image registration (see section 2.4) [35].

Sections were divided into six series. Every sixth thin section was processed for BDA histochemistry [36], producing a series of 74 sections covering all regions connected to M1. These sections were photographed under 0.5× magnification using a Nikon DXM1200F digital camera mounted on a Nikon E-800 microscope (image resolution = 7 µm/pixel, image size = 3840×3072 pixels). More than one photograph was needed to cover the entire section. Those component photographs were merged into a panoramic micrograph using Adobe Photoshop CS3 (San Jose, CA) and the background of each panorama was extended to a net image size of 6660×6660 pixels. The resulting stack of images defined a “standard micrograph space” which is the target space of all higher resolution microscopic data. Another series of sections was stained for Nissl substance to identify the cytoarchitectonic borders of M1 and other cortical regions connected to M1. Four series of remaining sections were used for other purposes.

### Micrograph-space to DTI-space Registration

The standard micrograph space was registered to the DTI image data using a multi-step procedure. First, every BDA-labeled section was down-sampled (to 256×256 pixels) and registered to the down-sampled photograph (256×256 pixels) of the corresponding tissue block using a 2D affine transformation followed by a 2D non-rigid transformation, semi-automatically calculated via the Thin-Plate Spline algorithm [37]. Next, all the down-sampled block photographs were assembled into a block volume and all the non-diffusion weighted MRI images were similarly stacked into a DTI volume. Then the block volume was registered to the DTI volume using a 3D affine transformation followed by a 3D non-rigid transformation automatically calculated via the Adaptive Bases Algorithm [38]. The multi-step registration described here was very similar to the registration procedure validated in an earlier study [39], which showed that the accuracy of the overall registration was approximately one MRI voxel (0.3 mm). The deformation fields produced by all the above registration steps were saved in order to transfer other data from the micrograph space (i.e., higher resolution microscopic data, described in section 2.5) into DTI space.

### Fiber and Density Distribution Map (DDM) Reconstruction

Our ultimate goal in this part of the study was to reconstruct histological and DTI-tractography-based DDMs in DTI space and render them on the same white-gray matter (WGM) interface. Prior to calculating DDMs, we reconstructed the 3D WGM interface in DTI space: the T2w image volume was rigidly registered to the DTI volume; white matter was segmented using a variational level set approach [40] on the aligned T2w images; the 3D outline of white matter was extracted to generate triangle meshes representing the WGM interface. In addition, the BDA-injection region and different cortical projection regions were segmented manually in the standard micrograph space based on architecture revealed by Nissl- and BDA-labeled sections [33] and then transferred to DTI space.

To resolve each individual BDA-labeled fiber, 4× micrographs (0.87 µm/pixel) covering the WGM boundary were acquired using a Nikon D1R1 digital camera mounted on a Nikon AZ100 M microscope. The BDA-labeled fibers were segmented by a series of morphological processes: top-hat filtering was performed to correct uneven illumination, global thresholding was implemented to extract fibers, and a set of statistical properties (e.g., area of object and ratio of perimeter to area) of each extracted object was calculated and used to remove the non-fiber objects. For each micrograph, points along the WGM boundary were selected manually and fit to a curve with 6 pixels (∼5 µm) width to represent the histological WGM boundary. Segmented fibers that overlapped the fitted boundary were extracted automatically. The centroids of the overlapping fibers were rigidly transferred to the standard micrograph space, slice by slice.

Next, the standard size micrograph (6660×6660 pixels) was divided into 256×256 square units and the number of centroids in each unit area, n_i,j_ (i,j = 1,2,…,256), was determined. These n_i,j_ values served as the intensity of the 2D DDM. The DDMs were transferred from micrograph space to block space then to DTI space using the deformation field associated with the registration procedure described earlier (see section 2.4). Immediately following each spatial transformation step, the Jacobian matrix of the corresponding deformation field was calculated and then used to compensate the density change caused by the raw geometric transformation. Finally, to facilitate visualizing the density information in 3D, we mapped these gray-matter-distributed densities onto the 3D WGM interface in DTI space. Thus, ideally, the value of a certain voxel on the 3D WGM interface in DTI space represented the number of BDA-labeled fibers whose centroids were located on the WGM boundary in high resolution micrograph space.

To identify the locations of BDA-labeled somas in the micrographic volume, we exhaustively plotted the centers of these somas and the outline of the brain tissue for each BDA-labeled section under a 6.3× objective of a Zeiss microscope equipped with the vector graphing software Igor Pro 2.0 (Wavemetrics, Inc., Lake Oswego, OR). Using Canvas 11 (ACD Systems, Victoria, British Columbia, Canada), each vector graph was converted to a bitmap image with resolution high enough to resolve any two adjacent soma markers. The bitmap image was rigidly aligned with the 0.5× micrograph of the corresponding BDA-labeled section by matching the outline of the brain tissue and then was cropped into a standard size image. Next, each labeled soma was segmented by simple thresholding and the centroid of the soma was extracted automatically; the procedures (including gridding, counting, and calculating, then transferring and compensating the DDM) were similar to those for processing BDA-labeled fiber data. Finally, we mapped these gray-matter-distributed densities onto the 3D WGM interface in DTI space: the density value of every gray matter voxel was added to the closest voxel located on the 3D WGM interface. Thus, ideally, the value for each voxel at the interface was the sum of the number of BDA-labeled somas located vertically (i.e., normal to the surface) beyond this voxel.

DTIStudio [41] software was used to perform tensor fitting and fiber tracking (using the FACT algorithm, [6]) over the whole brain (tensor fitting method: standard linear-fitting; tracking parameters: start fractional anisotropy, FA = 0.1, stop FA = 0.2 and stop angle = 70°). Streamlines penetrating both of the following two ROIs were selected: (i) the BDA-injection region transferred from the micrograph space and (ii) the WGM interface just underneath (i). The above deterministic tracking scheme is referred to as the ‘DS’ scheme below. The DDM for this tracking method was produced by counting the number of the streamlines passing through or terminating within the voxels at the WGM interface. In addition, the streamline terminals distributed within the cortical regions connected to M1 were also mapped onto the interface and then counted for each interface voxel to produce a DTI ‘streamline terminal’ DDM.

Probabilistic fiber tracking was performed with the FMRIB’s Diffusion Toolbox (FDT) v2.0 from the commonly-used FMRIB’s Software Library (FSL, http://www.fmrib.ox.ac.uk/fsl). First, the *bedpostx* command was run to calculate the distributions of diffusion parameters at each brain voxel using the Markov Chain Monte Carlo sampling method [10] with automatic relevance determination (ARD) weight assigned to 1 (default) and 0.5, respectively. The *probtrackx* command was used to generate probabilistic streamlines (using the following parameters: sample number = 10000, curvature threshold = 0.2, modified Euler streamlining = on, step length = 0.1 mm and distance correction = on). The BDA-injection region was used as the seed mask, the WGM interface underneath the injection region as the waypoint mask and the gray matter mask (excluding the injection region) as the termination mask. These probabilistic tracking schemes are referred to as ‘FSL1’ (ARD weight = 1) and ‘FSL2’ (ARD weight = 0.5), respectively. The voxel value in the resulting dataset represents the number of streamline samples passing through this voxel times the expected length of these samples. The probabilistic-tractography-derived DDM was generated by keeping the values of those voxels located at the WGM interface and setting the remaining voxels to zero. Again, the DDMs were rendered on the 3D WGM interface in DTI space.

### Quantitative Analysis

The number of DTI-tractography-derived streamlines has been proposed as a measure of cortico-cortical connectivity [18], based on the hypothesis that the number of streamlines is proportional to the number of axons connecting cortical regions. To test this hypothesis for inter-regional connectivity, the numbers of BDA-labeled fibers (*N_B_*) passing into cortical ROIs and the numbers of DTI streamlines (*N_D_*) projecting to the same ROIs were quantified. These data were fit to a linear model:
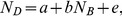
(1)where *a* and *b* are, respectively, the estimated intercept and slope of the regression line, and *e* is the error term. If *a* = 0 lay within the 95% of confidence interval of the intercept, then the data were fit to a second tier linear model with intercept equal to zero:



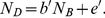
(2)The Pearson’s correlation coefficient of the fit, *r*, was calculated to indicate the degree of correlation between tractography and histology fiber data.

To verify the hypothesis in the case of intra-regional connectivity, we calculated the Pearson’s correlation coefficient, *r_p_*, between tractography and histology fiber DDMs within each cortical projection region on a voxel-by-voxel basis:
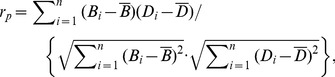
(3)where *B_i_* and *D_i_* are, respectively, the numbers of BDA-labeled fibers and DTI streamlines distributed in the *i* ^th^ voxel located at the WGM interface directly underneath this projection region, *n* is the total number of these voxels, and the bar represents the mean.

The original seed region covered the gray matter in the injection region, but it was found that DTI tractography was more effective when the seed region was intentionally extended one or two voxels into the white matter under the WGM interface (see below). To examine the effect of extending the depth, *d_w_*, of the seed region into the white matter on quantitative metrics, we calculated all correlations using seed masks with *d_w_* = 0, 1 and 2 voxels ( = 0, 0.3 and 0.6 mm).

In addition, since obtaining the DDM of BDA-labeled somas was less time-consuming, we studied the feasibility of using soma number to represent histological connectivity. We therefore calculated the relationship between numbers of somas and fibers across all projection regions. We also calculated the association between numbers of somas and numbers of DS streamline-terminals across all the projection regions.

### Fiber Orientation Comparison

To elucidate the reasons for discrepancy between tractographic and histological connectivity, we compared the tensor orientation with BDA-labeled fiber orientation in the high resolution micrograph space. Based on a multi-step registration procedure [39], the locations of diffusion tensors were transferred from the DTI space into the high resolution histological space and the tensor orientations were corrected based on the preservation of principal direction algorithm [42].

## Results

### M1-connected Cortices

BDA injections in the forearm representation area of the primary motor cortex reveal somatotopically distributed connections with the ipsi−/contralateral supplementary motor areas (iSMA/cSMA); ipsi−/contralateral anterior cingulate cortex (iAC/cAC); ipsi−/contralateral premotor cortex (iPM/cPM); ipsilateral M1 excluding the injection region (iM1ex); contralateral M1 (cM1); ipsi−/contralateral anterior parietal cortex (iPA/cPA); ipsi−/contralateral posterior parietal cortex (iPP/cPP); anterior part of upper bank of ipsilateral lateral sulcus –a combination of parietal ventral and rostral (iPVR) areas; posterior part of upper bank of ipsi−/contralateral lateral sulcus cortex–secondary somatosensory cortex (iS2/cS2). Labeling of ipsi−/contralateral prefrontal cortex (iPF/cPF) and contralateral PVR (cPVR) were not observed in this case. The above regions served as our regions of interest (ROIs). The borders of these ROIs were identified based on their cytoarchitectural features seen in Nissl preparations [43].

### Detection and Counting of Cross-interface BDA-labeled Fibers

**Figure 2:**
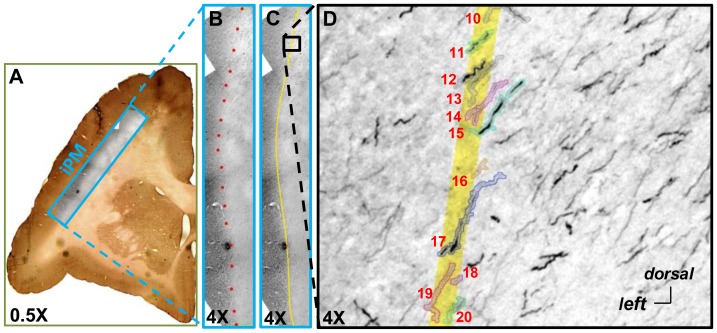
Pipeline for detecting and counting interface-crossing BDA-labeled fibers. Grayscale 4× micrograph (B) covering a specific ROI (iPM in this example), aligned to the corresponding 0.5× micrograph (A) in standard micrograph space. (B) shows manually drawn markers (red dots) used to identify the WGM boundary. (C) shows the fit of the markers to a continuous curve (yellow). (D) shows segmented BDA-labeled fibers (those with color contours) that touch the WGM boundary along with their numerical index (red numbers beside the fibers).

Figure 2 illustrates the pipeline for semi-automatic detection of cross-interface BDA-labeled fibers in the high resolution micrograph space. The WGM boundary (yellow curve shown in Fig. 2C) was estimated by fitting a series of manually-placed markers (red dots in Fig. 2B) on 4× micrographs covering a specific ROI. Interface-crossing fibers (fibers with color contours shown in Fig. 2D) were detected automatically by performing an “AND” operation on the fiber and boundary binary masks. Locations of the interface-crossing fibers were defined by the centroids of these fibers, and the number of cross-interface fibers was counted (red numbers in Fig. 2D). It was verified that shifting the WGM matter boundary towards white matter or gray matter by 50 pixels (∼44 µm) did not significantly change the number of interface-crossing fibers. The deviation of semi-automated counting from manual counting as the “gold standard” was also studied. Four to five sections for each ROI were randomly drawn for verification. The deviations for all the ROIs were in the range of 3%–5%.

### Tensor Fitting Results

**Figure 3:**
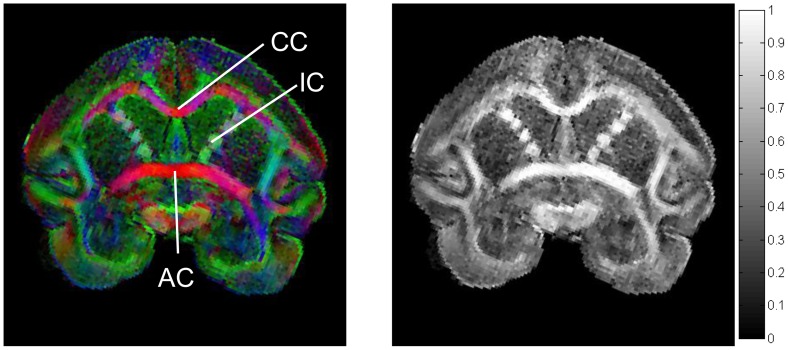
Maps of color-coded primary diffusion direction (red = Right/Left, green = Anterior/Posterior, and blue = Superior/Inferior) and scalar FA value (CC-corpus callosum, IC-internal capsule and AC-anterior commissure).

The DWI data were fit to single-tensors using DTIStudio. The primary diffusion direction and FA were calculated in each voxel. Figure 3 shows a color-coded map of primary diffusion direction and the FA scalar map of the same coronal slice.

### Correlation of Inter-ROI Connectivity Strength

**Figure 4:**
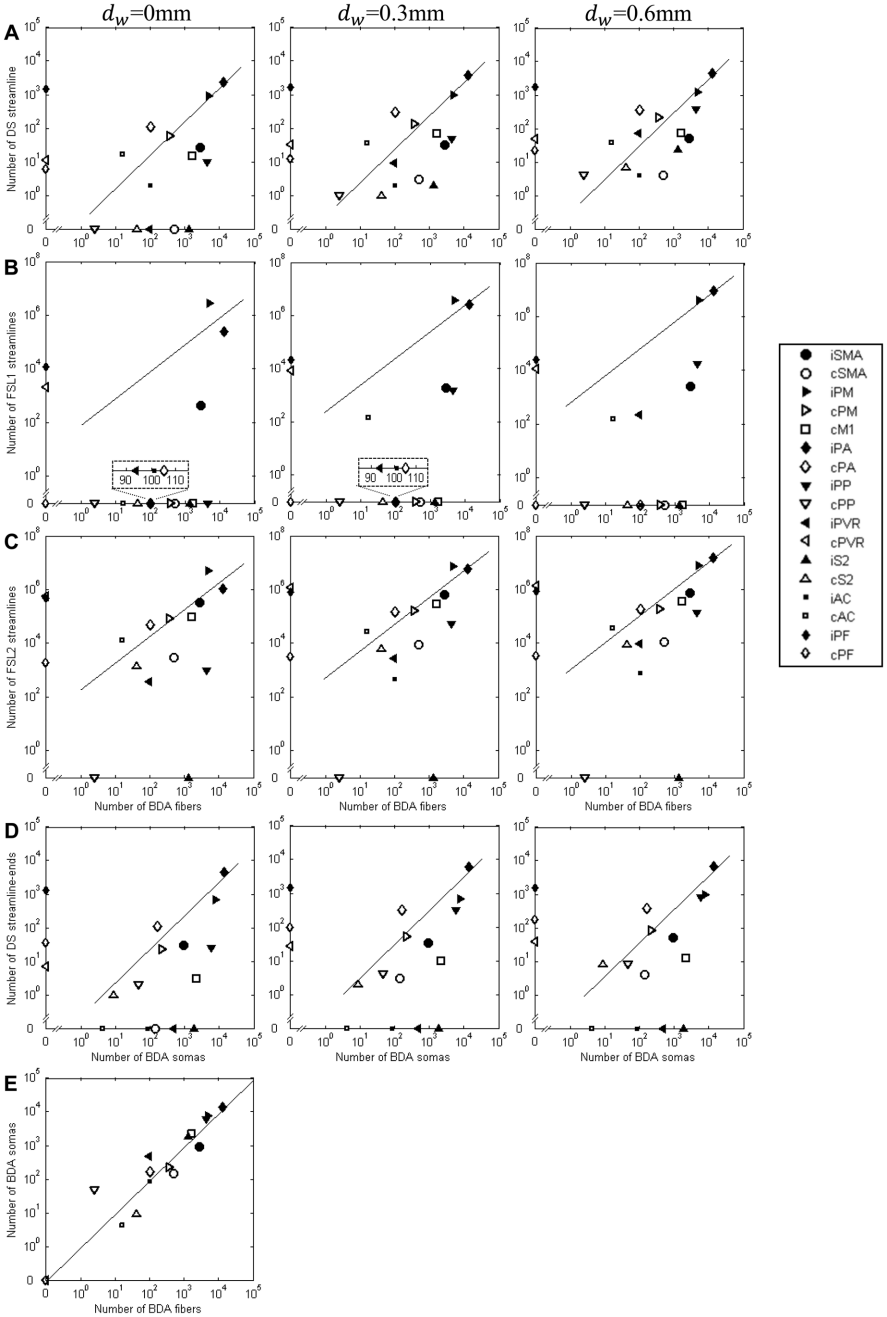
Relationship between tractography-histology variables as well as histology-histology variables. (A–C) show streamline vs. BDA-labeled fiber data; (D) shows streamline terminals vs. soma data; and (E) shows BDA-labeled soma vs. fiber data. DS (A and D), FSL1 (B) and FSL2 (C) schemes were used to obtain tractography-derived streamlines when *d_w_* is 0, 0.3 and 0.6 mm. Proportional relationships were fit based on least squares regression. The correlation coefficients (with corresponding *p* values) of the regressions are listed in Table 1.

**Figure 5:**
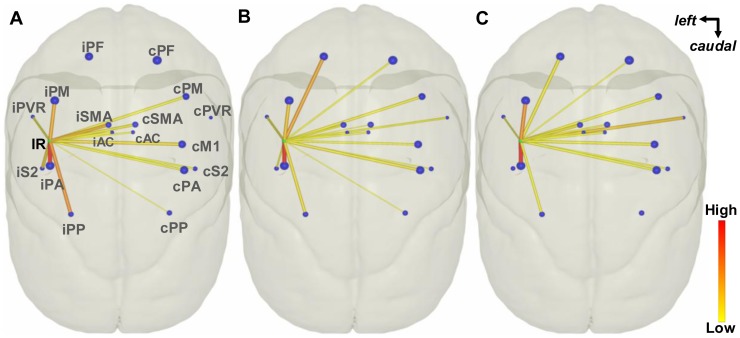
Dorsal view of the inter-regional connectivity backbones. (A), (B) and (C) show the BDA-labeled fiber, DS and FSL2 derived (*d_w_* = 0.6 mm) connectivity backbones, respectively. Green and blue nodes indicate the center of mass of the injection and individual projection regions, respectively. The radius of each node is scaled by the square root of the volumes of the corresponding region. The thickness of each edge represents the logarithmic connection strength and the color of the edge is coded according to connection weight (strength divided by volume of the projection region).

To test the hypothesis that the number of streamlines, *N_D_*, connecting two cortical regions is proportional to the number of anatomical fibers, *N_B_*, connecting those regions, we identified the number pair for connections between M1 and each ROI and plotted them in Fig. 4 as well as displayed them as connectivity backbones, shown in Fig. 5. The points lying on the horizontal axis (*N_D_* = 0) and vertical axis (*N_B_* = 0) in Fig. 4 indicate false negative and false positive connections, respectively. Approximately 75–98% of streamlines reached true positive ROIs for our predefined regions. By performing linear regression based on model (1) and calculating the 95% confidence interval for the intercept, we found that all nine confidence intervals included the origin. Therefore, we fit to the constrained linear model (2) to generate the regression lines in Fig. 4. The Pearson correlation coefficient *r* and corresponding *p* value for each case are shown in Table 1. Because the data span many orders of magnitude, the Pearson correlation coefficient (and corresponding *p*) is dominated by the largest values. For this reason, we also calculated the Spearman rank correlation coefficient, *r_s_*, and corresponding *p* value, which depends only on the relative rank of connection strengths measured by the two methods. The values of *r_s_* in Table 1 are quite low and do not reach statistical significance. To focus on the strongest connections, we chose the ten regions with the highest number of BDA-labeled fibers (i.e., number of BDA fibers>100) and calculated the Spearman rank correlation coefficient, (*r_s_*)_10_, and corresponding *p* value for this subset of the data.

**Table 1:** Pearson correlation (*r*) and Spearman rank correlation (*r_s_* and (*r_s_*)_10_) coefficients (with corresponding *p* values) for tractography-histology variables as well as histology-histology variables. Significant correlations are shown in bold.

	*d_w_* = 0 mm	*d_w_* = 0.3 mm	*d_w_* = 0.6 mm
	*r* = 0.75 (*p*<0.0005)	*r* = 0.82 (*p*<0.0001)	*r* = 0.86 (*p*<0.0001)
**BDA fibers vs. DS streamlines**	*r*_s_ = 0.30 (*p*<0.24)	*r*_s_ = 0.36 (*p*<0.16)	*r*_s_ = 0.45 (*p*<0.07)
	(*r*_s_)_10_ = 0.44 (*p*<0.21)	(*r*_s_)_10_ = 0.49 (*p*<0.15)	**(** ***r*** **_s_)_10_ = 0.67 (** ***p*** **<0.035)**
	*r* = 0.33 (*p*<0.20)	***r*** ** = 0.73 (** ***p*** **<0.0008)**	***r*** ** = 0.93 (** ***p*** **<0.0001)**
**BDA fibers vs. FSL1 streamlines**	*r*_s_ = 0.23 (*p*<0.38)	*r_s_* = 0.27 (*p*<0.30)	*r*_s_ = 0.29 (*p*<0.26)
	**(** ***r*** **_s_)_10_ = 0.74 (** ***p*** **<0.015)**	**(** ***r*** **_s_)_10_ = 0.86 (** ***p*** **<0.0014)**	**(** ***r*** **_s_)_10_ = 0.89 (** ***p*** **<0.0006)**
	*r* = 0.41 (*p*<0.098)	***r*** ** = 0.75 (** ***p*** **<0.0005)**	***r*** ** = 0.92 (** ***p*** **<0.0001)**
**BDA fibers vs. FSL2 streamlines**	*r*_s_ = 0.25 (*p*<0.34)	*r*_s_ = 0.35 (*p*<0.17)	*r*_s_ = 0.36 (*p*<0.15)
	(*r*_s_)_10_ = 0.61 (*p*<0.067)	**(** ***r*** **_s_)_10_ = 0.66 (** ***p*** **<0.044)**	**(** ***r*** **_s_)_10_** = **0.66 (** ***p*** **<0.044)**
	***r*** ** = 0.80 (** ***p*** **<0.0001)**	***r*** ** = 0.81 (** ***p*** **<0.0001)**	***r*** ** = 0.84 (** ***p*** **<0.0001)**
**BDA soma vs. DS streamline-** **ends**	*r*_s_ = 0.19 (*p*<0.47)	*r*_s_ = 0.23 (*p*<0.38)	*r*_s_ = 0.23 (*p*<0.37)
	**(** ***r*** **_s_)_10_ = 0.63 (** ***p*** **<0.050)**	**(** ***r*** **_s_)_10_ = 0.63 (** ***p*** **<0.050)**	**(** ***r*** **_s_)_10_ = 0.69 (** ***p*** **<0.026)**
**BDA fibers vs. BDA soma**	***r*** ** = 0.97 (** ***p*** **<1.0e-10)**		
	***r*** **_s_ = 0.95 (** ***p*** **<4.5e-9)**		

Comparison across the three tractography schemes with *d_w_* = 0 or 0.3 mm (distance of zero or one voxel) indicates that the linear correlations for the two probabilistic schemes are significantly lower than those of the deterministic scheme. When *d_w_* = 0.6 mm, however, the linear correlations for these three schemes are quite similar and deterministic tractography gave no false negative results. In addition, under any fixed *d_w_*, the FSL1 and FSL2 schemes have similar *r* value, but FSL2 has a smaller number of false negative ROIs than FSL1 does.

The influence of *d_w_* on the three tractography schemes, shown in the rows of Fig. 4A–C and first nine rows of Table 1 are different. As *d_w_* increases, the number of false negative ROIs decreases except for the FSL2 scheme. For the FSL1 and FSL2 schemes, as *d_w_* increases, the *r* value increases significantly. For the DS scheme, *r* value increases less strongly than for the FSL1 and FSL2 schemes.

The proportional relation between the number of BDA-labeled somas and the number of streamline-terminals, shown in Fig. 4D and Table 1 row 10 is similar to the relationship between BDA-labeled fibers and streamlines in Fig. 4A and Table 1 row 1, except the number of false negative ROIs were more for the soma-terminals case. The proportional relationship between BDA-labeled soma and fibers, shown in Fig. 4E and bottom 2 rows in Table 1, is statistically significant.

Fig. 5 shows the backbones of the BDA-labeled, DS and FSL2 derived inter-regional connectivity. There are no edges connecting IR to iPF, cPF and cPVR shown in Fig. 5A but there are edges between IR to those regions in Fig. 5BC, which indicates iPF, cPF and cPVR are false positive regions detected by DS and FSL2 with *d_w_* = 0.6 mm.

### Correlation of Intra-ROI Connectivity Spatial Distribution

**Figure 6:**
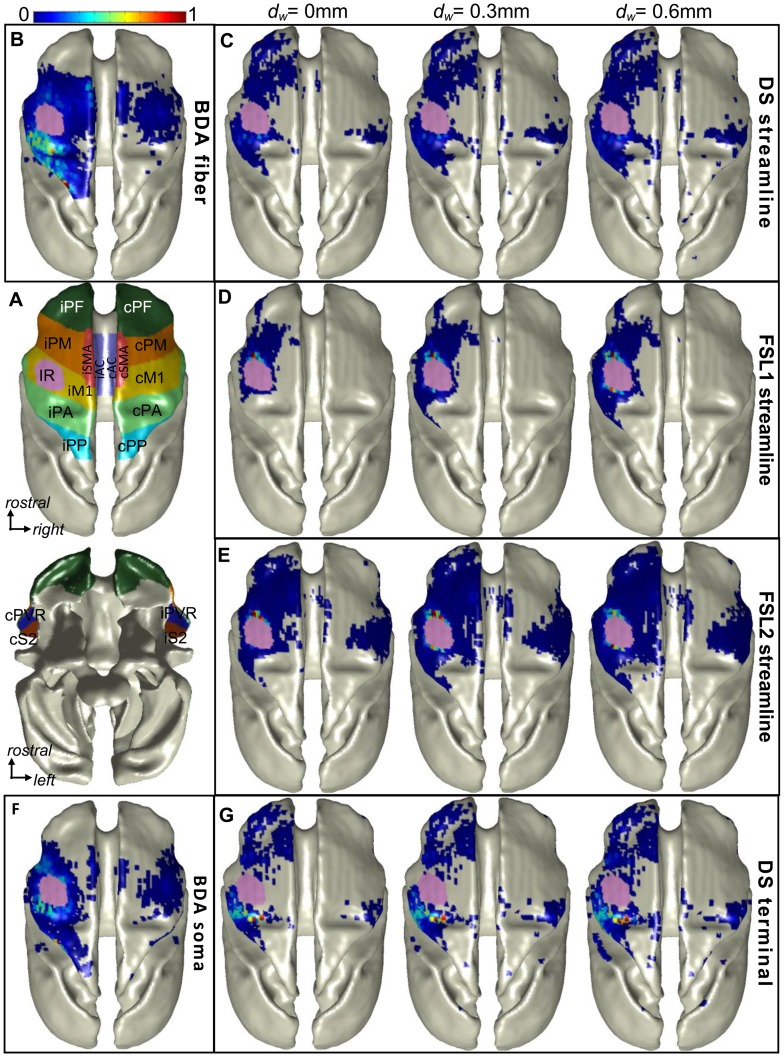
Dorsal view of 3D DDMs rendered on the WGM interface. (A) shows the territories of all the ROIs and the BDA injection region (upper-dorsal view; bottom-ventral view). (B) shows the BDA fiber DDM and (C–E) show respectively the streamline DDMs using DS, FSL1 and FSL2 tractography schemes (in rows) with different *d_w_* (in columns). (F) shows BDA soma DDM and (G) shows streamline terminal DDMs with different *d_w_* (in columns). Note that some hot spots in (B) are obscured in this view. All data are presented in Fig. 4 and the Tables.

To visualize the spatial distribution of fibers (or somas) as well as streamlines (or streamline-terminals) from the ROI scale down to the voxel scale, the density distribution of BDA somas, BDA interface-crossing fibers, DTI streamline-terminals and DTI interface-crossing streamlines were rendered on the 3D WGM interface in DTI space (Fig. 6). Comparison between Fig. 6B and Fig. 6C–E indicated that distribution patterns of BDA and DTI fibers (DS and FSL schemes) are quite different. Table 2 lists the quantitative evaluation of the agreement: the Pearson’s correlation coefficient, *r_p_*, of anatomical and DTI derived distributions in each individual ROI. Overall, the significant linear correlations for all the ROIs were lower than 0.5. The FSL2 scheme provided stronger linear correlations than FSL1 did, but comparable correlations to the DS scheme for most ROIs. The agreement in iPM is higher than the other projection regions due to higher sensitivity of DTI along this fiber pathway.

**Table 2:** Pearson’s correlation coefficients (with *p* values) of histological distributions and DTI tractography-derived distributions for all the projection regions.

d_w_ (mm)			iAC	iSMA	iPM	iM1	iPA	iPP	iPVR	iS2	cAC	cSMA	cPM	cM1	cPA	cPP	cPVR	cS2
**0**	BDA-DS fibers	r_p_	−.02	.01	**.35**	**.22**	**.41**	**.18**	−.00	.00	−.02	−.00	−.05	−.04	**.22**	.00	−.00	−.00
		p	.64	.86	.00	.00	.00	. 00	1	1	.65	1	.26	.32	.00	1	1	1
	BDA-FSL1 fibers	r_p_	.00	**.19**	**.39**	**.31**	**.12**	.00	−.00	.00	.00	−.00	−.00	−.00	−.00	.00	.00	−.00
		p	1	.00	.00	.00	,00	1	1	1	1	1	1	1	1	1	1	1
	BDA-FSL2 fibers	r_p_	−.02	**.24**	**.38**	**.34**	**.18**	−.00	−.02	.00	−.01	.04	−.06	.01	**.20**	.00	.00	.02
		p	.68	.00	.00	.00	.00	.94	.80	1	.79	.45	.14	.01	.00	1	1	.72
	BDA-DS somas	r_p_	.00	.07	**.34**	**.24**	**.33**	**.37**	−.00	.00	.00	−.00	.01	.07	.05	−.01	.00	**.59**
		p	1	.11	.00	.00	.00	.00	1	1	1	1	.89	.03	.09	.85	1	.00
**0.3**	BDA-DS fibers	r_p_	−.02	.02	**.33**	**.22**	**.43**	**.18**	−.02	−.04	−.02	−.04	−.04	−**.07**	**.23**	−.00	−.00	−.01
		p	.64	.67	.00	.00	.00	.00	.77	.35	.66	.44	.29	.04	.00	.94	1	.80
	BDA-FSL1 fibers	r_p_	.00	**.20**	**.43**	**.40**	**.15**	.08	−.00	.00	−.01	−.00	−.00	−.00	−.00	.00	.00	−.00
		p	1	.00	.00	.00	.00	.19	1	1	.87	1	1	1	1	1	1	1
	BDA-FSL2 fibers	r_p_	−.03	**.26**	**.43**	**.45**	**.20**	**.19**	−.03	.00	−.01	**.11**	−.06	.04	**.26**	.00	.00	−.03
		p	.58	.00	.00	.00	.00	.01	.67	1	.84	.02	.14	.28	.00	1	1	.55
	BDA-DS somas	r_p_	.00	.10	**.34**	**.24**	**.31**	**.24**	−.00	−.00	.00	−.02	−.00	.03	**.10**	−.01	.00	**.41**
		p	1	.04	.00	.00	.00	.00	1	1	1	.66	.95	.35	.00	.82	1	.00
**0.6**	BDA-DS fibers	r_p_	**.09**	.02	**.36**	**.26**	**.41**	**.13**	−.04	−.03	−.02	−.03	−.05	−.07	**.23**	−.01	−.00	−.02
		p	.05	.69	.00	.00	.00	.04	.57	.56	.65	.47	.26	.04	.00	.91	1	.62
	BDA-FSL1 fibers	r_p_	.00	**.22**	**.43**	**.44**	**.20**	**. 16**	−.01	.00	−.01	−.00	−.00	−.00	−.00	.00	.00	−.00
		p	1	.00	.00	.00	.00	.01	.88	1	.87	1	1	1	1	1	1	1
	BDA-FSL2 fibers	r_p_	.01	**.27**	**.45**	**.48**	**.21**	**.22**	−.04	.00	−.01	**.10**	−.06	.05	**.26**	.00	.00	−.02
		p	.83	.00	.00	.00	.00	.00	.57	1	.80	.03	.14	.13	.00	1	1	.60
	BDA-DS somas	r_p_	.00	.08	**.41**	**.29**	**.30**	**.32**	−.00	−.00	.00	−.02	−.01	.05	**.11**	−.02	.00	**.16**
		p	1	.09	.00	.00	.00	.00	1	1	1	.61	.86	.15	.00	.74	1	.00

Significant correlations are shown in bold, and correlation results for labeled somas are shown in italics.

We also investigated whether deeper seed regions could help to achieve better distribution similarity by increasing the probability that streamlines could bypass crossing fibers directly beneath the WGM interface. Results for *d_w_* = 0, 0.3 and 0.6 mm for each tractography scheme are shown in rows of Table 2. For the DS scheme, no significant trend in correlations was found as *d_w_* increased (*p*>0.99 in one-way ANOVA). For the FSL1 and FSL2 schemes, correlations for ipsilateral ROIs did not have statistically significant differences (*p*>0.75 and *p*>0.72) although they seemed to have subtly increasing trends as *d_w_* increased. No test was performed for contralateral ROIs, since the correlations were weak in those cases.

### Sources of Discrepancies between Histological and DTI Connectivity

**Figure 7:**
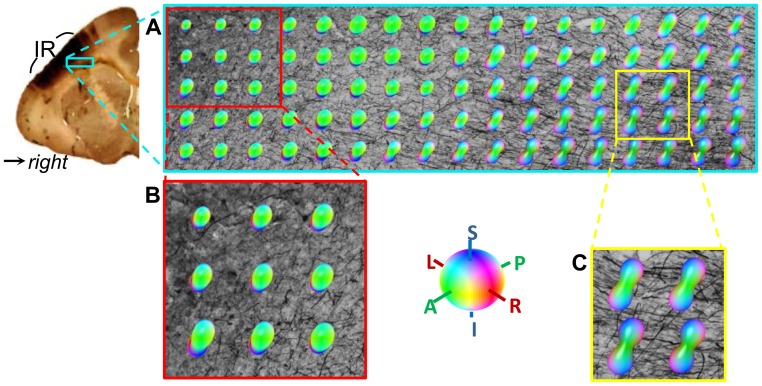
Three dimensional diffusion isosurfaces overlaid on BDA-labeled fibers in crossing fiber regions. Background of (A) is the high resolution BDA micrograph (4×) of the white matter under the injection/seed region. Diffusion isosurfaces calculated from the tensors are color-coded according to anatomical orientation and scaled in size by the local FA. (B) and (C) show details immediately under the injection region (IR) and in deeper white matter, respectively.

**Figure 8:**
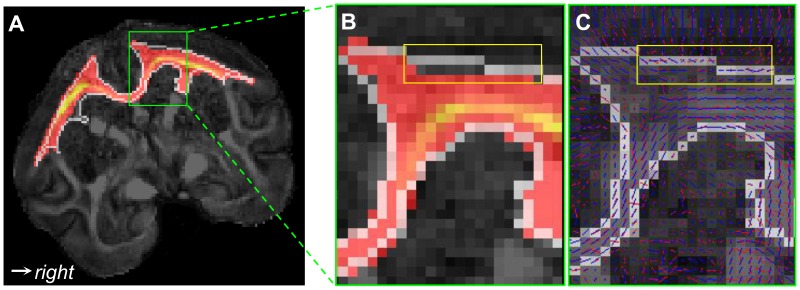
FSL2 outputs overlaid on a FA map. (A) Coronal slice showing the FSL2 density map (*d_w_* = 0.6 mm) superimposed on a grayscale FA map. The white curve labels the WGM interface. (B) and (C) show an enlarged region in the superior part of contralateral hemisphere. Blue and red lines in (C) represent dominant fiber orientations, estimated by the FSL *bedpost* tool.

The challenges facing diffusion MRI tractography are well known, in principle. Limitations due to image artifacts and noise [26], [44], [45], partial volume averaging [46], and angular resolution [47] have been studied both experimentally and theoretically. Partial volume averaging was an obvious source of error in this study, as illustrated in Figures 7 and 8. Although BDA-labeled fibers connecting the injection region to the contralateral hemisphere run left/right in the plane of Figure 7, the principal diffusion direction (corresponding to most fibers in these voxels) is oriented anterior/posterior just under the injection region (Fig. 7B) and superior/inferior in deeper white matter (Fig. 7C). The disagreement between the BDA fibers bound for the contralateral hemisphere and the dominant fiber orientation prevents most DTI streamlines from reaching the midline and the other hemisphere. In fact, the strong anterior/posterior orientation of tensors immediately under the injection region is partially responsible for the strong (false positive) connectivity to the ipsilateral prefrontal cortex (Fig. 6) measured with both the DS and FSL2 schemes.

Probabilistic tracking methods based on the multi-tensor model are predicted to be better able to resolve crossing fibers, but the histology vs. FSL fiber correlations (Table 2) are not significantly higher than the histology vs. DS fiber correlations in this study. Figure 8AB shows that FSL2 is also limited by partial volume averaging in this data set. The FSL2 streamlines do not reach the WGM interface under the contralateral PM cortex. Figure 8C displays the two fiber orientations estimated by the FSL2 scheme in each white matter voxel immediately underneath the contralateral PM cortex. The vast majority of axons in these voxels travel parallel to the WGM interface and strongly bias the FSL-estimated fiber orientations also to be nearly parallel to the WGM interface. All six FSL cases (two ARD values, three seed region depths) were subject to similar limitations (although it is possible that the estimated orientations could be more accurate at higher SNR and/or b-value [48]). In summary, partial volume averaging of diffusion tensor data is likely a major source of error in our cortical connectivity measurements for both deterministic and probabilistic tracking algorithms.

## Discussion

In this study, we evaluated the correlation of histology- and tractography-derived connectivity between M1 and 17 other cortical regions. In each case, histology and tractography density maps were compared both between (Fig. 4 and 5) and within (Table 2, Fig. 6) regions. To the best of our knowledge, this is the first work to make direct comparisons of cortico-cortical connectivity derived from histology and DTI tractography.

Our results show that DTI tractography was capable of detecting all the cortical regions anatomically connected to M1 (as in the DS scheme with *d_w_* = 0.6 mm), but also produced false positive connections to other regions (all schemes). In addition, the correlation of inter- and intra-ROI connection strengths indicates that the tractography schemes we used do not have uniform sensitivity to anatomical connections either across all ROIs or within a single ROI. Statistical analysis of Table 1 and visual inspection of Figure 4 support the conclusion that DTI tractography reliably identifies the regions with highest connectivity to M1. The Pearson correlation *r* (*p*<0.0001) was highly significant for all schemes with *d_w_* = 0.6 mm in Table 1, however this measure emphasizes the strongest connections. When regions with weaker M1 connectivity are included in the Spearman correlation test, DTI tractography is not able to determine the rank order reliably (non-significant Spearman *r_s_* for all schemes and seed depths). When the test is repeated with only the 10 regions most strongly connected to M1 included, the Spearman (*r_s_*)_10_ is highly significant. Hence, DTI tractography was not reliable in ranking the regions with weaker connectivity (i.e., number of BDA-labeled fibers smaller than 100).

The two tractography algorithms used in the study were not compared in a rigorously equivalent manner. For example, the seed volume for DTIStudio was the entire brain whereas FSL was seeded only in the injection region. These seeding strategies were chosen to match the way the algorithms are generally used, in practice. Our aim was less to make a head-to-head comparison of the two methods and more to assess the accuracy of DTI tractography as usually practiced.

The proportional relationship between the BDA-labeled somas and BDA-labeled fibers across all the ROIs was significant in our study, due to the reciprocal nature of motor cortex cortical connections (i.e., the comparable number of afferent and efferent nerve fibers for M1). However, other networks may not have reciprocal connections, and therefore the utility of BDA-labeled somas in validation studies is probably limited.

The tracer BDA was used in this study because it is transported in both anterograde and retrograde directions along axons leading to/from the injection site, which is analogous to the propagation of DTI streamlines from a seed region. Moreover, since BDA (in contrast to WGA-HRP [49]) does not diffuse much in brain tissue, the injected tracer was confined to the forearm M1 representation area. Most importantly, BDA provides highly sensitive and exquisitely detailed Golgi-like labeling of somas, axons and terminals [36], which better facilitates quantitative analysis of individual fibers than WGA-HRP and Mn^2+^ do. Although Mn^2+^ is an attractive tracer for validation studies because it is directly visible on MR [30], the specificity of Mn^2+^ for fiber pathways is limited, especially for our purpose of quantitative comparison based on counting individual axons.

We found approximately 75–98% of streamlines reached true positive ROIs for our predefined regions (section 3.4). These percentages represent the sensitivities of the nine tracking strategies (DS, FSL1 and FSL2 schemes using *d_w_* = 0, 0.3 and 0.6 mm), which are comparable to the 80% sensitivity measured by Seehaus et al [50] in post mortem human temporal lobe. The pathways measured in that paper were seeded in white matter voxels and terminated in cortical injection site(s), whereas our experiment aimed to assess corticocortical connectivity, which means valid fibers were required to touch both injection and target cortical regions.

There are several limitations to the study. First, only connections to the forearm region of M1 were quantified–measurements of the accuracy of DTI connectivity to this region may not generalize to other areas in M1 or to other cortical networks. Second, BDA was injected at approximately 1 mm intervals covering the forearm region (Fig. 1). This interval was chosen because the tracer spreads in the tissue about 0.5 mm from the needle location and the goal was to tag all axons in the region. However, it is possible that uptake of the tracer was not uniform over the forearm area while the distribution of tractography seed points was uniform. Hence, it is possible that the BDA and seed point distributions were not identical in the injection region, which could bias the results toward those locations within the region where BDA uptake was higher. In addition, in the radial direction, the BDA injection was not strictly confined to gray matter, but extended slightly into the white matter immediately beneath the cortex, which means that fibers of passage underneath the injected cortex may have taken up BDA as well. Thus we used seed regions that extended one or two voxels (*d_w_* = 0.3 and 0.6 mm) into the subcortical white matter to match the injection. Third, imperfect micrograph-to-DTI spatial registration may introduce bias in DDMs. Although the overall error in our registration procedure was likely on the order of 1 voxel ( = 0.3 mm) [39], the local error close to the GWM-interface was more difficult to control and estimate because there were fewer apparent features to be captured as landmarks. Fourth, the MRI acquisition had limited SNR (∼30) and number of diffusion directions (n = 31), which likely affected sensitivity to small fiber components. However, these are typical parameters for many DTI experiments, including *in vivo* human studies. Finally, this study used two approaches to tractography (deterministic and probabilistic), implemented in two of the most commonly used analysis packages (DTIStudio and FSL). However, the results may not apply to other algorithms. The framework for comparison to histology used in this study could be applied to other algorithms.

Probabilistic tractography schemes were expected to provide stronger correlations than deterministic tractography schemes due to higher sensitivity to non-dominant pathways. However, our results show no significant difference in correlations between these methods. This might be due to the limitations of our DWI acquisition, since 32 diffusion directions only allow FSL to resolve crossing angles more than 60° within a voxel [48]. Additionally, due to partial volume averaging, the termination mask used in FSL tended to stop some streamlines which otherwise would travel along the gray matter interface and then stop at locations father away from seed mask. This phenomenon is shown as a ‘hot color’ ring surrounding the injection region in Fig. 6E.

The results of this study show that connectivity measured by DTI tractography is strongly correlated with anatomical connectivity when measured on the scale of major cortical regions (Fig. 4). At a finer scale (i.e., within regions), the DTI DDMs are somewhat less reliable (Table 2). This implies that as cortical parcels are subdivided to achieve higher resolution in connectivity maps, the reliability of those maps may decrease dramatically, due to inaccuracies in streamline terminal location. Our results suggest that interference from strongly anisotropic bundles that cross pathways of interest tends to bias DTI connectivity measurements. Such bias would likely be consistent across repeated scans and across individuals, if the fiber geometry was consistent. Hence, reproducibility of DTI connectivity measurements is not, in and of itself, an indication of reliability. More accurate connectivity measurements will rely on detection of fibers that comprise a small minority of the total fiber population within a voxel. This may be possible with High Angular Resolution Diffusion Imaging (HARDI) methods, although the sensitivity of HARDI to small fiber populations has not been studied extensively. Improvements in modeling fiber orientation distributions and tracking fibers of interest through ambiguous crossing regions will be critical to increasing the accuracy of diffusion MRI measurements of cortical connectivity.

Finally, the squirrel monkey replicates much of the macroscopic structure of the human nervous system, yet it is possible that the human brain has more complex microstructure. Thus some caution is advised extrapolating our results to DTI measurements in the human brain.

## Conclusions

DTI tractography is capable of detecting true connectivity for most or all cortical regions with anatomical connections to M1 in the squirrel monkey, but may also produce false positive connections. Deterministic tractography can provide inter-regional connectivity measures as accurate as probabilistic tractography under some situations. DTI tractography is not uniformly sensitive to connection strength either across or within connecting regions. A major source of discrepancy between histological and tractography-based connectivity is the presence of crossing fibers, which may prevent streamlines from reaching the correct bundle from the cortical seed region or prevent them from leaving a strongly anisotropic white matter bundle to find the correct cortical terminals.

## References

[1] Basser PJ, Mattiello J, Lebihan D (1994) MR diffusion tensor spectroscopy and imaging. Biophysical Journal 66: 259–267. 8130344 10.1016/S0006-3495(94)80775-1 PMC1275686

[2] Stejskal EO, Tanner JE (1965) Spin diffusion measurements: Spin echoes in the presence of a time-dependent field gradient. Journal of Chemical Physics 42: 288–292.

[3] Beaulieu C (2002) The basis of anisotropic water diffusion in the nervous system - a technical review. NMR in Biomedicine 15: 435–455. 12489094 10.1002/nbm.782

[4] Mori S, Zhang JY (2006) Principles of diffusion tensor imaging and its applications to basic neuroscience research. Neuron 51: 527–539. 16950152 10.1016/j.neuron.2006.08.012

[5] Basser PJ, Pajevic S, Pierpaoli C, Duda J, Aldroubi A (2000) In vivo fiber tractography using DT-MRI data. Magnetic Resonance in Medicine 44: 625–632. 11025519 10.1002/1522-2594(200010)44:4<625::aid-mrm17>3.0.co;2-o

[6] Mori S, Crain BJ, Chacko VP, van Zijl PCM (1999) Three-dimensional tracking of axonal projections in the brain by magnetic resonance imaging. Annals of Neurology 45: 265–269. 9989633 10.1002/1531-8249(199902)45:2<265::aid-ana21>3.0.co;2-3

[7] Poupon C, Clark CA, Frouin V, Regis J, Bloch I, et al (2000) Regularization of diffusion-based direction maps for the tracking of brain white matter fascicles. Neuroimage 12: 184–195. 10913324 10.1006/nimg.2000.0607

[8] Westin CF, Maier SE, Mamata H, Nabavi A, Jolesz FA, et al (2002) Processing and visualization for diffusion tensor MRI. Medical Image Analysis 6: 93–108. 12044998 10.1016/s1361-8415(02)00053-1

[9] Conturo TE, Lori NF, Cull TS, Akbudak E, Snyder AZ, et al (1999) Tracking neuronal fiber pathways in the living human brain. Proceedings of the National Academy of Sciences of the United States of America 96: 10422–10427. 10468624 10.1073/pnas.96.18.10422 PMC17904

[10] Behrens TEJ, Woolrich MW, Jenkinson M, Johansen-Berg H, Nunes RG, et al (2003) Characterization and propagation of uncertainty in diffusion-weighted MR imaging. Magnetic Resonance in Medicine 50: 1077–1088. 14587019 10.1002/mrm.10609

[11] Lazar M, Alexander AL (2005) Bootstrap white matter tractography (BOOT-TRAC). Neuroimage 24: 524–532. 15627594 10.1016/j.neuroimage.2004.08.050

[12] Parker GJM, Haroon HA, Wheeler-Kingshott CAM (2003) A framework for a streamline-based probabilistic index of connectivity (PICo) using a structural interpretation of MRI diffusion measurements. Journal of Magnetic Resonance Imaging 18: 242–254. 12884338 10.1002/jmri.10350

[13] Hagmann P, Thiran JP, Jonasson L, Vandergheynst P, Clarke S, et al (2003) DTI mapping of human brain connectivity: statistical fibre tracking and virtual dissection. Neuroimage 19: 545–554. 12880786 10.1016/s1053-8119(03)00142-3

[14] Friman O, Farneback G, Westin CF (2006) A Bayesian approach for stochastic white matter tractography. IEEE Transactions on Medical Imaging 25: 965–978. 16894991 10.1109/tmi.2006.877093

[15] Kubicki M, McCarley R, Westin CF, Park HJ, Maier S, et al (2007) A review of diffusion tensor imaging studies in schizophrenia. Journal of Psychiatric Research 41: 15–30. 16023676 10.1016/j.jpsychires.2005.05.005 PMC2768134

[16] Parker GJM, Luzzi S, Alexander DC, Wheeler-Kingshott CAM, Ciccarelli O, et al (2005) Lateralization of ventral and dorsal auditory-language pathways in the human brain. Neuroimage 24: 656–666. 15652301 10.1016/j.neuroimage.2004.08.047

[17] Oishi K, Zilles K, Amunts K, Faria A, Jiang HY, et al (2008) Human brain white matter atlas: Identification and assignment of common anatomical structures in superficial white matter. Neuroimage 43: 447–457. 18692144 10.1016/j.neuroimage.2008.07.009 PMC2586008

[18] Hagmann P, Kurant M, Gigandet X, Thiran P, Wedeen VJ, et al (2007) Mapping Human Whole-Brain Structural Networks with Diffusion MRI. Plos One 2: 9. 10.1371/journal.pone.0000597 PMC1895920 17611629

[19] Singer C (1952) Vesalius on the human brain. Oxford: Oxford University Press.

[20] Marchi V, Algeri G (1885) Sulle degenerazioni discendenti consecutive a lesioni sperimentali in diverse zone della corteccia cerebrale. Riv Sper Freniatria Med Legal 11: 492–494.

[21] Waller A (1850) Experiments on the section of the glossopharyngeal and hypoglossal nerves of the frog and observations of the alterations produced thereby in the structures of their primitive fibers. Phil Trans R Soc A 140: 423–429.

[22] Nauta WJH (1993) Some early travails of tracing axonal pathways in the brain. Journal of Neuroscience 13: 1337–1345. 8463822 10.1523/JNEUROSCI.13-04-01337.1993 PMC6576740

[23] Morecraft RJ, Ugolini G, Lanciego JL, Wouterlood FG, Pandya DN (2009) Classic and contemporary neural tract tracing techniques. Diffusion MRI: from quantitative measurement to in-vivo neuroanatomy: Academic press. 274–307.

[24] Vercelli A, Repici M, Garbossa D, Grimaldi A (2000) Recent techniques for tracing pathways in the central nervous system of developing and adult mammals. Brain Research Bulletin 51. 10.1016/s0361-9230(99)00229-4 10654576

[25] Pautler RG, Silva AC, Koretsky AP (1998) In vivo neuronal tract tracing using manganese-enhanced magnetic resonance imaging. Magnetic Resonance in Medicine 40: 740–748. 9797158 10.1002/mrm.1910400515

[26] Anderson AW (2001) Theoretical analysis of the effects of noise on diffusion tensor imaging. Magnetic Resonance in Medicine 46: 1174–1188. 11746585 10.1002/mrm.1315

[27] Hofer S, Frahm J (2006) Topography of the human corpus callosum revisited - Comprehensive fiber tractography using diffusion tensor magnetic resonance imaging. Neuroimage 32: 989–994. 16854598 10.1016/j.neuroimage.2006.05.044

[28] Hubbard PL, Parker GJM (2009) Validation of tractography. In: Johansen-Berg H, Behrens TEJ, editors. In: Diffusion MRI: from quantitative measurement to in-vivo neuroanatomy: Academic press. 353–376.

[29] Lawes INC, Barrick TR, Murugam V, Spierings N, Evans DR, et al (2008) Atlas-based segmentation of white matter tracts of the human brain using diffusion tensor tractography and comparison with classical dissection. Neuroimage 39: 62–79. 17919935 10.1016/j.neuroimage.2007.06.041

[30] Dyrby TB, Sogaard LV, Parker GJ, Alexander DC, Lind NM, et al (2007) Validation of in vitro probabilistic tractography. Neuroimage 37: 1267–1277. 17706434 10.1016/j.neuroimage.2007.06.022

[31] Dauguet J, Peled S, Berezovskii V, Delzescaux T, Warfield SK, et al (2007) Comparison of fiber tracts derived from in-vivo DTI tractography with 3D histological neural tract tracer reconstruction on a macaque brain. Neuroimage 37: 530–538. 17604650 10.1016/j.neuroimage.2007.04.067

[32] Hagmann P, Cammoun L, Gigandet X, Meuli R, Honey CJ, et al (2008) Mapping the structural core of human cerebral cortex. Plos Biology 6: 1479–1493. 10.1371/journal.pbio.0060159 PMC2443193 18597554

[33] Stepniewska I, Preuss TM, Kaas JH (1993) Architectonics, somatotopic orgnizations, and ipsilateral cortical connections of the primary motor area (M1) of owl monkeys. Journal of Comparative Neurology 330: 238–271. 7684050 10.1002/cne.903300207

[34] D’Arceuil HE, Westmoreland S, de Crespigny AJ (2007) An approach to high resolution diffusion tensor imaging in fixed primate brain. NeuroImage 35: 553–565. 17292630 10.1016/j.neuroimage.2006.12.028

[35] Toga AW, Ambach KL, Schluender S (1994) High-resolution anatomy from in-situ human brain. Neuroimage 1: 334–344. 9343583 10.1006/nimg.1994.1018

[36] Reiner A, Veenman CL, Medina L, Jiao Y, Del Mar N, et al (2000) Pathway tracing using biotinylated dextran amines. Journal of Neuroscience Methods 103: 23–37. 11074093 10.1016/s0165-0270(00)00293-4

[37] Bookstein FL (1989) Principal warps: thin-plate splines and the decomposition of deformations. IEEE Transactions on Pattern Analysis and Machine Intelligence 11: 567–585.

[38] Rohde GK, Aldroubi A, Dawant BM (2003) The adaptive bases algorithm for intensity-based nonrigid image registration. IEEE Transactions on Medical Imaging 22: 1470–1479. 14606680 10.1109/TMI.2003.819299

[39] Choe A, Gao Y, Li X, Compton K, Stepniewska I, et al (2011) Accuracy of image registration between MRI and light microscopy in the ex vivo brain. Magnetic Resonance Imaging 29: 683–692. 21546191 10.1016/j.mri.2011.02.022 PMC3100355

[40] Li C, Huang R, Ding Z, Gatenby C, Metaxas D, et al.. (2008) A variational level set approach to segmentation and bias correction of images with intensity inhomogeneity. MICCAI. 1083–1091. 10.1007/978-3-540-85990-1_130 PMC2782702 18982712

[41] Jiang HY, van Zijl PCM, Kim J, Pearlson GD, Mori S (2006) DtiStudio: Resource program for diffusion tensor computation and fiber bundle tracking. Computer Methods and Programs in Biomedicine 81: 106–116. 16413083 10.1016/j.cmpb.2005.08.004

[42] Alexander DC, Pierpaoli C, Basser PJ, Gee JC (2001) Spatial transformations of diffusion tensor magnetic resonance images. IEEE Transactions on Medical Imaging 20: 1131–1139. 11700739 10.1109/42.963816

[43] Preuss TM, Stepniewska I, Kaas JH (1996) Movement representation in the dorsal and ventral premotor areas of owl monkeys: A microstimulation study. Journal of Comparative Neurology 371: 649–675. 8841916 10.1002/(SICI)1096-9861(19960805)371:4<649::AID-CNE12>3.0.CO;2-E

[44] Lazar M, Alexander AL (2003) An error analysis of white matter tractography methods: synthetic diffusion tensor field simulations. Neuroimage 20: 1140–1153. 14568483 10.1016/S1053-8119(03)00277-5

[45] Huang H, Zhang JY, van Zijl PCM, Mori S (2004) Analysis of noise effects on DTI-based tractography using the brute-force and multi-ROI approach. Magnetic Resonance in Medicine 52: 559–565. 15334575 10.1002/mrm.20147

[46] Alexander AL, Hasan K, Lazar M, Tsuruda JS, Parker DL (2001) Analysis of partial volume effects in diffusion-tensor MRI. Magnetic Resonance in Medicine 45: 770–780. 11323803 10.1002/mrm.1105

[47] Cho K-H, Yeh C–H, Tournier J-D, Chao Y-P, Chen J-H, et al (2008) Evaluation of the accuracy and angular resolution of q-ball imaging. Neuroimage 42: 262–271. 18502152 10.1016/j.neuroimage.2008.03.053

[48] Behrens TEJ, Berg HJ, Jbabdi S, Rushworth MFS, Woolrich MW (2007) Probabilistic diffusion tractography with multiple fibre orientations: What can we gain? Neuroimage 34: 144–155. 17070705 10.1016/j.neuroimage.2006.09.018 PMC7116582

[49] Kobbert C, Apps R, Bechmann I, Lanciego JL, Mey J, et al (2000) Current concepts in neuroanatomical tracing. Progress in Neurobiology 62: 327–351. 10856608 10.1016/s0301-0082(00)00019-8

[50] Seehaus AK, Roebroeck A, Chiry O, Kim D-S, Ronen I, et al (2013) Histological Validation of DW-MRI Tractography in Human Postmortem Tissue. Cerebral Cortex 23: 442–450. 22345356 10.1093/cercor/bhs036 PMC3584953

